# Giving justice to listening: exploring the impact of a novel dialogical approach to mental wellbeing on healthcare staff

**DOI:** 10.3389/fpsyg.2025.1576278

**Published:** 2025-08-12

**Authors:** Isaac Nsiah, Cornelia Junghans, Henock B. Taddese, Anna Dentschuk, Amanda Bueno de Mesquita, Matthew Ball

**Affiliations:** ^1^School of Public Health, Imperial College London, London, United Kingdom; ^2^Healthcare Central London, London, United Kingdom; ^3^Central North West London Health Trust, London, United Kingdom; ^4^Humane Clinic, Adelaide, SA, Australia

**Keywords:** Just Listening, mental health, wellbeing, health workers, mental health training

## Abstract

**Introduction:**

A recent survey among health care workers in the United Kingdom revealed that over 75% experienced a mental health problem in the past year, with 42% reporting chronic stress. Poor mental wellbeing adversely affects patient outcomes and places a financial burden on the NHS. To address this issue, a novel dialogical approach called “Just Listening” (JL), originally used in community settings in Australia, has been introduced to train frontline workers in the United Kingdom. JL aims to maintain human-to-human connection and offers intentional listening. Evidence shows it to be effective in improving mental wellbeing of members in the community. It is unknown how effective this approach is in improving mental wellbeing when applied to training healthcare staff. This study aims to explore perceptions of JL training amongst a variety of health professionals and how JL can be utilised to improve mental health and wellbeing in a health care context.

**Methods:**

We employed qualitative methodology, gathering data through semi-structured interviews with healthcare workers who had undergone JL training in Westminster. We analysed 17 semi-structured interviews with a broad range of staff who had undergone training several months prior. We used self-determination theory and transformational learning theory as guiding conceptual frameworks. In addition, we applied thematic analysis to post-course feedback gathered from participants to explore if views of the training differ between the time of the training (post course feedback) and several months later when the interviews were carried out. Analysis was conducted using Nvivo 14.

**Results:**

The study revealed five key themes: the power of listening, shifting perspectives, empowerment and professional development, enhanced work relationships and implementation. Themes across the immediate post-training feedback and structured interviews overlapped. Participants reported that JL training improved team and client relationships as well as their personal lives, leading to improved mental wellbeing. However, system constraints were identified as a challenge, emphasizing the need for support from senior management and policymakers to further enable its use.

**Discussion:**

This was the first study to evaluate JL in the United Kingdom and in a healthcare setting. Our findings show that there was an overwhelming impression of positive impact of the training on the professional and personal lives of healthcare staff. This study contributes to the growing body of knowledge on mental health interventions for healthcare professionals, providing evidence that can inform future practices and policies. JL presents a viable model to improve staff wellbeing. Our findings reinforce growing calls for a culture shift towards human connection.

## Highlights

• The burden of stress on mental health in healthcare workers is significant, resulting in sickness absence and poor patient care.
• Dialogical and relational approaches therapeutic in nature such as active listening are evidenced to result in better wellbeing, better patient outcome and a better workplace climate.
• Just Listening is a novel relationally therapeutic approach to mental wellbeing that was shown to improve mental wellbeing of members of the community and volunteers in Australia.
• This study demonstrates that JL can lead to improved mental wellbeing among a variety of healthcare professionals in the United Kingdom.

## Background

Health care workers are usually faced with significant stress due to the demands of their work, caused often by staff shortages, heavy workloads, and the pressure to sustain high-quality patient care (Rink et al., 2023). This adversely affects both their physical and mental wellbeing (Darzi, 2024). A recent report highlights a deeply disengaged NHS workforce with “distressingly high” sickness absence and “low staff morale” (Darzi, 2024). Globally, burnout, stress and mental ill-health are major issues affecting healthcare workers (Medscape, 2021; NHS Charities Together, 2024): 42% of physicians reported burnout while 47% noted its adverse effect on their personal lives in the United States (Medscape, 2021). Similarly, a recent survey among National Health Service (NHS) staff in the United Kingdom revealed that over 75% experienced a mental health problem in the previous year, with 42% reporting chronic stress (NHS Charities Together, 2024). This contributes to increased sickness absence as well as presenteeism, which has significant cost implications (Ravalier et al., 2020; NHS England, 2024a). Staff sickness absences in the NHS due to work-related stress and mental ill-health costs the organization up to £400 million annually (NHS Employers, 2024; Rimmer, 2018). In 2023, NHS sickness absence reached a record high, with poor mental health accounting for more than a quarter of all absences (NHS Charities Together, 2024).

Poor mental wellbeing among healthcare staff has also been linked to compromising patient care and safety standards (Hall et al., 2016; Motluk, 2018; Chana et al., 2015). In extreme cases, chronic stress, burnout and mental ill-health have led to suicide among healthcare workers (Stehman et al., 2019; Troglio da Silva and Neto, 2021), with reports of up to 23% times higher risk of suicide compared to the national United Kingdom average in some healthcare staff (Causer et al., 2022). This further entails profound adverse impacts on surviving healthcare colleagues, who often experience intense emotional responses including grief, trauma, guilt, confusion and fear (Causer et al., 2022). It is therefore imperative to equip healthcare staff with the skills to manage both their patients‘ mental health and their own and in doing so contribute to the wellbeing of their colleagues. The NHS health and wellbeing framework recognises the importance of health and wellbeing of all NHS staff (NHS England, 2024b). Several training modalities exist, including but not limited to emotional logic, resilience training, coaching, counseling, and active listening.

There is growing evidence from other studies to show the impact of listening interventions in the work setting (Kluger and Itzchakov, 2022). In a recent scoping review, listening was identified as crucial for achieving social justice in social work practice (Aadam et al., 2024). Listening skills were recommended as essential practice rather than additional skill. When listening is practiced sincerely, honestly, and genuinely, it can help professionals meet their standards and aid in the recovery of those they support, fosters increased trust and leads to better outcomes (Aadam et al., 2024). When practitioners improved their empathic listening, there was an improvement in overall patient satisfaction and outcomes (Howick et al., 2018). Several studies demonstrated the influence of listening in the workplace on work performance and professional relationships (Hinz et al., 2021; Kriz et al., 2021; Kluger et al., 2024). Customer service employees who were trained in listening became less anxious when they had to speak to difficult customers because of their improved listening skills (Itzchakov, 2020). Higher perception of listening was associated with lower levels of burnout in students (Pines et al., 2002).

### Justice to listening as a novel approach to mental wellbeing

Just Listening (JL) is a novel dialogical approach to improving mental wellbeing that has recently been introduced to frontline staff in the London borough of Westminster with a population of approximately two hundred and five thousand (205,000) people with varying health needs. The need for an approach that simultaneously enables better care for patients and better self-care for staff arose with the introduction of a new healthcare-related role in the United Kingdom: the Community Health and Wellbeing Worker programme (CHWW) (Junghans et al., 2024), whereby trained lay people are employed to undertake community outreach proactively to around 120 households each within deprived areas as part of the wider Primary Care team, to support people with profound needs. JL has been demonstrated as a model that support both lay and clinically trained workers in the Just Listening Community in Australia (Ball et al., 2022).

### Just Listening (JL)

Just Listening‘s novelty lies in emphasizing the concept of “giving justice to a person’s story by listening,” listening in a just way (Ball et al., 2022; Just Listening Community, 2024). Improved mental wellbeing is almost a by-product of connection enacted in the process of listening. Justice in this context means that the listener allows the story to unfold without offering judgement, opinion, solutions or inserting their own similar stories (Ball et al., 2022; Just Listening Community, 2024). In a nutshell, the listener is not listening with the intention to fix or respond, but to provide space and empathy to allow the speaker to hear themselves out loud (Ball et al., 2022; Just Listening Community, 2024). This also enables the listener to listen to their own inner voices and impulses to respond (Just Listening Community, 2024). It is in the context of a person being supported to hear their own story, through the process of Just Listening, that the individual can hear their own experiences in a new way, a way that creates opportunity to process the impact of events in their lives, be these work or personal difficulties. This is described by Family medical Practitioner Lew Mehl Madrona: “Listening provides what is typically a rare opportunity to really speak and experience ourselves being heard, perhaps allowing ourselves to be heard for the first time…this is why listening is so powerfully therapeutic” (Mehl-Madrona, 2015).

Unlike similar active listening or coaching approaches that are unidirectional and goal-oriented, often focused on improving practitioner performance in relation to the client only, Just Listening (JL) is inherently mutual from the outset (Just Listening Community, 2024). JL reframes the act of listening not as a skill to be applied to another, but as a relational practice that engages both speaker and listener in a shared human encounter (Just Listening Community, 2024). Rather than placing the listener in a hierarchical role of helper, JL acknowledges the listener’s own subjectivity and the unique emotional resonance of what is heard. This creates space for the listener to remain attuned not only to the speaker’s narrative but to their own internal responses, cultivating self-awareness, reflexivity, and the recognition of when they too need to be heard. In this way, JL decentralizes intervention and recenters connection, where listening becomes a mutual act of witnessing in turn respects both persons’ needs and vulnerabilities (Just Listening Community, 2024).

The origin of the name JL can be traced to the book by Gans (2001). JL focuses on providing a just process when listening to individuals experiencing emotional or mental distress, putting aside judgement (Gans, 2001). JL does not involve any formulation, plan, or intervention and places emphasis on the listener taking responsibility for their own actions and responses, rather than seeking to bring change by intervention (Ball et al., 2022). JL is relationally but not medically therapeutic and aims to enhance human connection between the speaker and the listener on a level. The principles of JL are 4-fold: (1) listen with the intention to offer justice to a person’s story (2) focus responses on the story of the person you are listening to, (3) resist the urge to fix (listen for longer) and (4) slow down: be aware of your thoughts and intentions (Ball et al., 2022).

Just Listening was first pioneered at the Humane Clinic in Australia in a community based setting as alternative crisis response to traditional medical responses to mental health crisis which may involve sectioning, medicating or sedating, and shown to be an effective response to human distress, with those who visited the centre in distress experiencing relief by the end of their JL session (Ball et al., 2022). Volunteers trained to offer JL also reported positive effects on their own mental health and wellbeing, noting that the training empowered them to support individuals in distress within their own families, workplaces, and the broader community (Ball et al., 2022). However, the report also highlighted that early on in the process of listening to members of the community, staff doing the listening recognised the need to be listened to in turn to recognise the experience of listening to others and maintaining a sense of self and freedom from vicarious trauma or burnout (Ball et al., 2022). The process of the listener being listened to, supports the central tenet of the community based model that any person could be the listener or the storyteller, as such the value of listening could be channelled towards the wellbeing of both. This work in a community setting in Australia (Ball et al., 2022) inspired the move to train healthcare staff in JL to improve their own mental wellbeing and by extension the wellbeing of their clients.

Just Listening challenges dominant psychiatric narratives and emphasizes the value of listening with justice, meaning listening in ways that honour power, voice, context, and truth and prioritise human connection. The three conceptual frameworks underpinning Just Listening are Power Threat Meaning Framework (PTMF) (Johnstone and Boyle, 2018), Suicide Narratives (Ball et al., 2020) and Dissociachotic (Ball and Picot, 2023). We set out below how they underpin the overarching principle of Justice to Listening:

Power Threat Meaning Framework (PTMF) developed by Johnstone and Boyle (2018) is a radical alternative to traditional psychiatric diagnosis. It asks: What has happened to you? (Power) How did it affect you? (Threat) What sense did you make of it? (Meaning) What did you have to do to survive? (Responses) (Johnstone and Boyle, 2018). Just Listening draws on PTMF to challenge the idea of individual pathology. Instead of “What’s wrong with you?” (the biomedical model), Justice to Listening insists on hearing the person’s story in context: It demands that professionals recognise systemic and relational abuse, trauma, racism, classism and colonialism as structural powers (Johnstone and Boyle, 2018). Listening with justice means acknowledging the threats created by these powers. Rather than framing behaviours as symptoms, JL encourages seeing them as survival strategies, which is central to respectful and ethical listening. Understanding someone’s meaning-making is central to honouring their voice and lived reality and supports any individual to make meaning in the process of storytelling: It is important to remember that storytelling and meaning-making are universal human capacities (Johnstone and Boyle, 2018; Ball et al., 2022).

Suicide Narratives (Ball et al., 2020) as a concept explores how people make sense of suicidality not as an outcome of a damaged brain and faulty thought process, but as a deeply human response to pain, oppression and meaning-loss. It reframes suicide away from “risk to be managed” toward “story to be heard.” Using the structure of the Maastricht approach to hearing voices (Moskowitz and Corstens, 2007), Suicide Narratives provides a functional framework to embed psychosocial understanding and responses to suicide risk, consistent with NICE guideline on Self-harm: assessment, management and preventing recurrence (NG225) that places the imperative on a psychosocial approach over a risk prediction model (National Institute for Health and Care Excellence [NICE], 2022).

Dissociachotic is a neologism or hybrid term developed by Matthew Ball, combining dissociation and psychosis (Ball and Picot, 2023). It reframes psychosis not as a broken brain, but as an extreme form of dissociation – a response to contexts of overwhelming trauma, loss or oppression and other bad life situations (Moskowitz et al., 2019). JL challenges pathologizing frameworks of schizophrenia or psychosis: Hearing voices, delusions, or altered states are reframed as deeply meaningful responses to trauma or existential crises (Corstens et al., 2018). Dissociachotic experiences can be understood to be communicative if we are willing to listen without interruption so that a person can share the narrative behind the expression. Thus, this framework underpins JLs call for justice-oriented listening: where the goal is not control or correction, but deep witnessing and co-regulation as described by Mosher (1999).

Just Listening draws on these frameworks with the recognition that extreme states such as psychosis and suicidality as well as any issues and difficult emotions people may grapple with in daily life can be effectively addressed through the transformative power of listening as the therapy. No matter how objectively extreme a response can be, JL represents active human to human engagement that fosters understanding, human connection, and healing through the process of human-to-human relationship (Ball et al., 2022). JL aligns with the principles of each theory by focusing on the individual‘s experiences and the meanings they attach to them, creating a compassionate and supportive environment. JL empowers the speaker to construct meaningful narratives that promote new awareness and understanding of previously unheard stories, and respects the autonomy of the speaker by listening without imposing interpretations (Ball et al., 2022). By adopting a JL approach, the listener creates a context for a person to share their story. The listener demonstrates mutuality by witnessing the speaker’s unique personal narrative, validating their experiences by trusting that the person will develop new understanding of their experiences when offered a just experience of listening (Mehl-Madrona, 2015).

In summary, JL introduces a relationally non-medical therapeutic approach, which emphasizes human connection and has the potential to positively impact staff wellbeing. Current dialogical approaches within the NHS are primarily therapeutic in nature (Tribe et al., 2019). This study aims to address the gap of evidence of such an approach by evaluating the experience of JL-trained healthcare staff in Westminster, some of which have received active listening training, coaching training and other mental health training, offering valuable insights into how JL can be integrated into healthcare settings to enhance staff mental wellbeing.

## Materials and methods

Just Listening was introduced to CHWWs and other healthcare staff in Westminster as one of the tools to equip staff to manage the mental ill-health of their clients as well as their own mental wellbeing from 2022. JL training included 16–18 h online training spread over a period of 4–6 weeks, some face-to-face training and regular check-in sessions. The training itself was centred in praxis, with teaching of the theory components delivered in the sessions, but dominated by practical application of listening in 1-2-1, smaller and bigger groups. A total of 120 staff were trained in JL over the period of 2 years.

Qualitative methodology was used to evaluate the impact of JL on the professional and personal lives of healthcare staff in Westminster. We combined the self-determination theory (SDT) (Patrick and Williams, 2012) and the transformative learning theory (TLT) (Anand et al., 2020) as guiding conceptual frameworks. SDT focuses on three basic psychological needs: autonomy, competence, and relatedness, which are essential for motivation and psychological wellbeing (Patrick and Williams, 2012). Good listening helps to satisfy these three needs specified in the SDT (Van Quaquebeke and Felps, 2018; Patrick and Williams, 2012). It offers a view of how professional development interventions can affect both the professional environment and personal lives by enhancing psychological wellbeing (Kluger et al., 2024). TLT emphasizes the process of perspective transformation through critical reflection on assumptions and beliefs (Anand et al., 2020). These offer the necessary conceptual tools to systematically study changes in professional and personal domains resulting from the JL training. The study utilized both primary and secondary data analysis of qualitative information to assess the long-term impact of JL training on the professional and personal lives of healthcare staff in Westminster. The primary outcome measure was participants’ views and perceptions regarding the impact of JL on their work and personal experiences.

Participants were selected using a purposive sampling technique to identify frontline staff who underwent the JL training. Formal recruitment emails were sent to frontline staff trained in JL. Each email included a participant information sheet which provided a comprehensive description of the research study, including details about participants’ rights to anonymity, data confidentiality, the right to withdraw. Once individuals agreed to participate, a consent form was emailed to them, before proceeding to book for the interview. To capture a range of perspectives, the study included participants from various roles who had undergone JL training. Ethical approval for this study was obtained from the Imperial College Research and Ethics Committee (ICREC): ID 7080202.

Primary data were gathered through semi-structured interviews conducted online using open-ended questions. This enabled the collection of rich data that allowed participants to freely express themselves. The interview guide was informed by the literature and the guiding conceptual frameworks. During the interviews, the researcher adapted the questions in the interview guide based on the responses from the interviewees to ensure flexibility and relevance. Seventeen interviews were conducted and each lasted between 30 and 45 min. Audio recording and transcriptions were done concurrently during the interview using Microsoft Teams. Transcripts were securely saved and accessible only to the researchers.

Secondary data were sourced from anonymous post-course feedback collected from participants immediately following their JL training. This data was also obtained with the help of the social care lead in Westminster. In all, twenty forms were retrieved. This comprised of open-ended questions where participants have given responses on how they perceived the JL training and its possible impact. Transcripts and post-course feedback were thoroughly reviewed. Any identifiable information was removed from interview scripts prior to uploading onto NVivo 14 software. This was to ensure confidentiality. The qualitative data was coded and analysed using NVivo version 14 to conduct a thematic analysis. Separate reflective thematic analysis was done on the primary and secondary data to elicit any differences in emerging themes between them. The six step approach by Braun and Clarke (Wilson et al., 2022) was followed during the thematic analysis and the process was iterative.

### Researchers’ reflexivity and positionality

Researchers throughout the study were aware of their positionality and how that could impact the study. All researchers had a background in healthcare and were aware of the stress and poor mental wellbeing experienced by healthcare staff. The primary researcher had neither undergone the JL training, nor used it before, so had no first-hand experience with it. This made him objective and impartial in his analysis of the training. He was also not known to participants prior to the study, so there was no lopsided power dynamics at play during interviews which enabled participants to freely express their views without any fears. The researchers’ positionalities therefore had a mix of both “emic perspective” (insider’s view) and “etic perspective” (outsider’s view) during the study (Seikkula et al., 2006; Olmos-Vega et al., 2023; Wilson et al., 2022). This enabled deep professional understanding and interaction as well as objective analysis. An interpretivist epistemology guided the entire research process, acknowledging the researchers’ positionalities as influencing factors on the research outcomes (Byrne, 2022). Three of the authors (CJ, AD, ABM) are trained in JL. Their feedback for the initial study was excluded from analysis and they were not part of the interview process. MB developed JL and is practising and teaching it on a daily basis. Throughout the study, the researchers maintained an “empathetic neutrality” stance, conscientiously monitoring their personal impact on the data while upholding transparency and impartiality toward the subject matter. This was to increase the validity and reliability of the research findings.

## Results

For primary data analysis 17 participants were recruited: nine Community Health and Wellbeing Workers (CHWW), four Health and Wellbeing Coaches (HWC), two General Practitioners (GP), and two Social Prescribers (SP). The proportion of type of front-line workers was representative of all categories of healthcare staff trained in JL, ensuring that all views were represented in the study.

All participants had their JL training more than 5 months prior to the study, with 41% of them trained more than a year ago. This allowed exploration of participants’ views about JL several months after the training. The details of the participants are shown in Table 1.

**TABLE 1:** Summary of participants for semi-structured interviews.

Participant code	Role	Period since JL training
IP 1, 2, 3, 4, 6, 7, 8, 9	CHWW	5 months
IP 5	CHWW	2 years
IP 10	SP	2 years
IP 11	SP	1 year
IP 12	HWC	6 months
IP 13, 14, 15	HWC	1 year
IP 16	GP	1 year
IP 17	GP	6 months

### Thematic analysis results

Five major themes and 17 sub-themes were identified (Table 2). The themes from the analysis of the primary interview data were similar to secondary data collected from post-course feedback with no new or different themes. This showed that time elapsed since training did not materially change how participants felt about it. Each theme was mapped to a domain of the theoretical frameworks adopted for this study.

**TABLE 2:** Themes and sub-themes linked to their theoretical framework domain.

Themes	Sub-themes	Theoretical framework domain
The power of listening	Foundation for dialogue, Creating safe spaces, Justice in listening, Increased self-awareness	Autonomy
Shifting perspectives	Culture change, Enhancing human connections, Transformation of organizational norms	Perspective transformation
Empowerment and professional development	Enhanced professional insight, Enhanced communication skills, Long-term wellbeing enhancements, Ongoing training needs	Competence
Enhanced work relationships	Team cohesion and collaboration, Improved personal relationships, Stress reduction	Relatedness
Implementation of JL	Appraisal of the training process, Challenges in full implementation, Leadership commitment	Critical reflection

### Theme 1: the power of listening

Across the board, all participants recognised listening as a powerful tool at the core of effective communication. Yet, they noted that in everyday interactions, people often fail to truly listen. Instead, they may rush to respond or offer solutions, which inhibits deep, empathetic engagement. Many participants felt that JL could offer a transformative solution to this issue. Of the 17 participants, 15 shared their personal testimonies on how JL had made a significant difference. One participant said:

“The training has been incredibly helpful. I’ve tested it with many people, and it’s fascinating how powerful JL is. The impact on mental health and wellbeing is amazing—people’s responses are profound when they feel truly listened to.” (IP 17, GP)

Just Listening was seen as the foundation for other dialogical approaches, with several participants advocating for its inclusion in training for other communication models. They suggested it could be beneficial for healthcare staff to start with JL as an introductory tool before moving on to more complex methods like open dialogue. As one participant, currently training in open dialogue, explained:

“Just Listening should be part of the foundational training–before diving into open dialogue, it’s essential to grasp the power of listening.” (Post-course feedback, HWC)

Crucially, JL allows people to share their thoughts freely, without fear of judgment. This safe space fosters authentic communication and enhances mental wellbeing. For healthcare workers themselves, this process doesn’t just benefit patients—it’s also an opportunity for staff to be heard. As one participant described,

“Just Listening gave me the opportunity to release a burden I’d carried for years. It was the space to express myself without fear, and just being heard helped me find clarity and a solution.” (Post-course feedback, SP)

The safety of authentic dialogue was also seen as vital in preventing crisis situations, including suicidality. A participant working in a high-pressure mental health environment shared:

“I’m struck by JL’s potential for suicide prevention. It works intuitively and powerfully. In my setting, we deal with high suicide rates—JL has a profound impact on our ability to connect with people and save lives.” (IP 17, GP)

Beyond its immediate effects, JL also served as a reminder for participants to slow down and truly listen to both patients and colleagues, shifting their approach to communication and fostering more meaningful connections.

“This training made me realize how often I’d rush to respond. Now, I’m much more mindful–whether with patients or colleagues. I’m genuinely listening, and it’s improved my relationships.” (IP 3, CHWW)

### Theme 2: shifting perspectives

A resounding call for change emerged from the data, with participants stressing the need for a fundamental shift in perspective toward mental wellbeing. They advocated for a culture shift that would embrace JL, not just as a technique, but as a crucial part of transforming healthcare interactions. As one participant put it:

“JL training is the reset we need in healthcare. It teaches us to listen first, not to focus on what the system can or can’t do. It leads to a cultural shift in how we approach care.” (IP 1, CHWW).

Just Listening was seen as more than just an effective communication tool; it was a reminder to re-prioritize human connections that are often overlooked in the hustle of healthcare. This emphasis on human connection–both within the workplace and in personal lives–was seen as essential for fostering mental health and wellbeing.

“This training has taught me that it’s not just about communication – it’s about truly connecting. JL makes that possible.” (Post-course feedback, CHWW)

Participants also expressed the need to transform the NHS system, which they felt often treats individuals as statistics rather than people. They envisioned a future where patient-centred care and personal connections would drive job satisfaction and improve the overall wellbeing of both staff and patients.

“In the NHS, we often treat patients as numbers, not people. This disconnect is damaging–not only does it affect our work, but it fractures our own sense of humanity. JL could be the bridge that reconnects us all.” (Post-course feedback, GP)

### Theme 3: empowerment and professional development

The empowerment that came from JL training was a recurring theme. Participants felt more equipped to listen, not just to others, but also to themselves. This newfound awareness of the power of listening translated directly into improved communication skills and a deeper connection with both patients and colleagues.

“Just Listening has opened up a new way of understanding my work. It’s not just about listening to my clients, but also understanding where I am in the emotional landscape of my work. It’s enhanced my ability to truly listen and engage.” (Post-course feedback, SP)

For many, JL training didn’t just affect their professional practice–it enhanced their personal relationships as well. One participant shared:

“This skill has made me a better communicator, not only at work but in my personal life too. It’s become a global wellbeing tool that benefits everyone.” (IP 8, CHWW)

Another noted that JL had visibly improved their work relationships and job satisfaction, offering a deeper sense of fulfilment in their role.

“Adopting JL in my practice has transformed the way I consult with clients. I feel more connected to my work and my clients, and it’s made me a happier, more fulfilled practitioner.” (Post-course feedback, GP)

### Theme 4: enhanced work relationships

The integration of JL into workplace dynamics was frequently cited as a major catalyst for improved team cohesion and collaboration. Several participants reported that JL check-in sessions within teams had strengthened relationships, leading to less stress and better teamwork. One participant described the positive impact within their team:

“The JL check-ins have created stronger bonds between us. We’ve become more cohesive, and the strength of our team has grown. These small human connections are what make us stronger.” (IP 1, CHWW)

Most participants felt that JL, when practiced in teams, led to better interpersonal relationships, which directly improved work performance and patient outcomes.

“By just listening to my colleagues and clients, I’ve seen how it transforms relationships. People feel heard, and this creates a stronger connection that really improves how we work.” (IP 12, HWC)

Just Listening was also seen as a crucial tool in reducing stress, especially in high-pressure settings. One participant shared how JL had become a key part of their team’s stress management strategy:

“Just Listening has been incredibly helpful in managing stress within our team. It’s an essential tool that allows us to offload our burdens and be present for our patients.” (IP 13, HWC).

### Theme 5: implementation of JL

While the benefits of JL were universally acknowledged, participants were clear that its successful implementation requires careful consideration. They stressed that JL should complement, not replace, existing communication tools in the healthcare setting.

The majority of participants had undertaken online training, with a few opting for face-to-face sessions. Though opinions varied, many felt the training was valuable, with some acknowledging the need for a safer, more structured approach to difficult topics like abuse and suicide. One participant said:

“There wasn’t any trigger warnings, and I feel like when they talked about suicide and abuse, it came unexpected. I know it did affect some of my colleagues when they were talking about abuse, particularly domestic and sexual abuse. So, I think that could have been done in a safer manner.” (IP 11, SP)

Despite its potential, some participants expressed concerns over the feasibility of fully implementing JL in settings with time constraints, like GP surgeries. However, they suggested it could still be used effectively in brief interactions, such as, at the start of a consultation, or during team check-ins to foster connection and emotional release.

“Due to time constraints, JL may not always be feasible during patient consultations, but it works well at the start of sessions. The key is choosing moments to implement it–those small, mindful moments can make a big difference.” (IP 17, GP)

Other challenges in full implementation included the need for ongoing training and the fact that not all staff had received JL training, especially in GP settings. Participants recommended that training be expanded to include all staff and be reinforced regularly.

“Not everyone gets it. The key to success is making sure all staff are trained so they can fully appreciate and practice JL.” (IP 16, GP)

Finally, leadership commitment was seen as crucial to the widespread adoption of JL. Participants in roles with supportive leadership described how regular JL check-ins had become ingrained in their team culture, reinforcing its benefits.

“If senior leaders experienced JL firsthand, they would see its value. I believe that would push for broader implementation across the system.” (IP 16, GP)

## Discussion

Our study showed that JL training has positively impacted trained healthcare staff professionally and in their personal lives. Healthcare staff have become more aware of the importance of empathic listening and self-awareness of their own responses and have enhanced their communication skills both with colleagues and clients. This, they believe, will lead to long-term improvement of their mental health and wellbeing. Through JL, participants felt that there has been improvement in their team collaboration and bonding through regular JL check-ins. For JL to be well implemented within the current NHS, participants felt that a culture change and a shift in perspectives towards prioritisation and maintenance of human-to-human connections was needed. Despite the positive impact from the JL training, there were some challenges that could affect full implementation of JL. Time constraints in attending to clients is one challenge that was identified. Also, staff struggled to incorporate JL in the context of their patient and client interactions and felt clients required more than JL. It was therefore imperative that every case is assessed individually to see which of the dialogical approaches will be best.

The findings from the study were consistent with previous insights into the impact of dialogical approaches and listening training on mental health and wellbeing (Quirk et al., 2018; Itzchakov, 2020). Our study confirmed that when people are genuinely listened to, they can gain new understanding in their narrative. Participants recognised the competence that JL training has given them, similar to that found in the quasi-experiments by Itzchakov (2020), where customer service workers trained in listening felt less anxious and more confident when they had to interact with difficult customers. Participants felt they have better working relationships with clients and colleagues after this training similar to Kluger et al. (2024) evidencing the positive impact of listening on work relationships.

Another value in JL mentioned by participants was its role in stress reduction with the majority of participants in the study mentioning that their work was stressful (NHS Charities Together, 2024). This role of JL in stress reduction was consistent with the findings of the cross-cultural study by Pines et al. (2002) among social care students who felt their levels of stress and burnout reduced after they had been trained in listening.

Participants were of the view that even though the current transactional nature of the NHS has a place, there needs to be a shift in perspective toward prioritizing human connection. Both employers and employees have a role to play, and JL can encourage these relational interactions and human connections.

Even though the training process was appraised as excellent there were some challenges that affected full implementation of JL and required adaptation in different practice settings. One such challenge was time constraints. GPs usually have limited time in attending to clients, so felt unable to use JL in its pure form. This issue of time constraints was also a barrier mentioned in the qualitative study by Quirk et al. (2018), when researchers sought to assess the facilitators and barriers to the implementation of health and well-being interventions within the NHS.

In summary, Just Listening (JL) offers a distinctive and coherent foundation for understanding and alleviating stress in healthcare professionals. Through its underpinning frameworks JL rejects pathologizing or diagnosticating models of distress, instead centring the value of narrative, meaning-making, and relational connection. Crucially, it legitimises the subjective experience of stress, not by externally categorising what qualifies as trauma, but by affirming that distress takes many forms and deserves to be heard with compassion, curiosity, and without expressing judgements. JL also blurs the conventional boundaries between personal and professional stress, recognising that these experiences are rarely compartmentalised in practice (Rink et al., 2023). Rather than reinforcing a hierarchical distinction between practitioner and patient, JL elevates both to the level of equal, human participants in a shared moment of meaning-making. In this way, JL offers not only a practice but a shift in paradigm, from doing to others, to being with them.

### Strengths and limitations

This is the first ever study to evaluate the impact of JL among healthcare workers. The use of both primary and secondary data for this study made it unique, as it allowed us to gauge the sustainability of impact on trained healthcare staff, and how JL principles were applied in practice. Secondly a variety of healthcare staff trained in JL were included to capture a wide range of perspectives and improve the generalizability of the results. It ensured a holistic picture was obtained about the impact of JL in Westminster. Most participants had received training in coaching, active listening and other mental health training prior to JL, allowing reference and comparison, although this was not an explicit aim of the study. Similarly, although post-course feedback and interview data were not matched by participant, themes in primary and secondary research were overlapping, indicating consistency of the impact of training. The opt-in process for participant recruitment may have skewed the results towards stronger views on JL, be it negative or positive.

Lastly, more CHWW were interviewed compared to the other categories of healthcare staff. This had the potential to affect the quality of data collected, as the views of the CHWW may overshadow that of the other categories of trained staff. However, data obtained from the CHWW did not differ from the other cadre of healthcare staff interviewed. The responses were similar across all the categories of staff. Hence, even if equal numbers of the different categories of healthcare staff were interviewed, the results are likely to remain unchanged.

### Policy implications

The findings from this study highlight the significant potential of JL in enhancing the mental health and wellbeing of healthcare staff. When practiced within healthcare settings, JL is likely to foster improved personal relationships, strengthens teamwork, and reduce stress levels. These are bound to contribute to increased job satisfaction and ultimately better patient outcomes. Additionally, by reducing stress and improving wellbeing, JL can decrease sickness absence, leading to cost savings for the NHS. The JL training provided frontline staff with essential skills in listening and mental wellbeing management, which are particularly beneficial given that many clients present with complex emotional and psychological issues. This makes JL a promising dialogical approach that policymakers should explore further to improve the wellbeing of both staff and clients. Expanding JL training beyond Westminster could reveal its impact on a larger scale. Incorporating JL into the induction programs for new healthcare workers could further equip them with crucial listening skills from the outset. Finally, managers should encourage their staff who have been trained in JL to apply these skills consistently and create opportunities for their use in practice.

### Future research

This study has qualitatively explored the impact of JL on healthcare staff, a quantitative study that can measure impact using various scales such as self-activation measures, wellbeing and service parameters such as sickness absence would add to the body of evidence. The corollary of improved wellbeing of staff is improved client interaction. However, further studies could measure direct impact on clients and patients, as well as impact on team work and productivity. Finally, results from our study have hinted at a positive impact of healthcare staff’s personal and professional relationships, this could be formally explored in further research.

In conclusion, this study sought to explore the impact of a novel dialogical approach to mental wellbeing on healthcare staff. It is the first study to evaluate JL in the healthcare setting, whereby findings have demonstrated numerous ways that JL has impacted healthcare staff who have been trained in it. The human-to-human connection that JL offers leads to improved personal and professional relationships which can increase job satisfaction and ultimately promote better patient outcomes. If JL can be adopted by policy makers and incorporated into the current NHS, it could contribute significantly to wellbeing and a culture shift towards relational work.

## Data Availability

The raw data supporting the conclusions of this article will be made available by the authors, without undue reservation.
